# Cross-Sectional Trajectories of Social Cognition in Later Life: Exploring Emotion Perception, Theory of Mind, and Emotional Empathy

**DOI:** 10.1093/arclin/acaf022

**Published:** 2025-03-09

**Authors:** Amy L Jarvis, Stephanie Wong, Michael Weightman, Benjamin Simmonds, Hannah A D Keage, Gail Robinson

**Affiliations:** Justice and Society Unit, University of South Australia, Magill 5072, South Australia, Australia; Justice and Society Unit, University of South Australia, Magill 5072, South Australia, Australia; College of Education, Psychology and Social Work, Flinders University, Bedford Park 5042, South Australia, Australia; Faculty of Health and Medical Sciences, University of Adelaide, Adelaide 5005, South Australia, Australia; College of Medicine and Public Health, Flinders University, Adelaide 5042, South Australia, Australia; School of Psychology, University of Adelaide, Adelaide 5005, South Australia, Australia; Justice and Society Unit, University of South Australia, Magill 5072, South Australia, Australia; School of Psychology, The University of Queensland, St Lucia 4067, Queensland, Australia; Queensland Brain Institute, The University of Queensland, St Lucia 4067, Queensland, Australia

**Keywords:** Social cognition, Theory of mind, Emotional recognition, Empathy

## Abstract

**Objective:**

The social cognitive abilities of emotion perception, cognitive theory of mind (ToM), affective ToM, and emotional empathy change across adulthood. Few existing studies have examined the performance of a single social cognitive domain in later life, with no known studies having examined all four abilities together. Although it is well understood how non-social cognitive performance changes with age, and this has helped inform diagnostic methods for age-related disorders, relatively little is known about typical age-related social cognitive performance in later life. The current study aimed to investigate the association between age and social cognitive performance within a sample of healthy midlife to older adults.

**Method:**

This cross-sectional study examined emotion perception using the Mini-SEA Facial Emotion Recognition Test, cognitive and affective ToM using The Shortened Awareness of Social Inference Test—Short Form, and emotional empathy using the Interpersonal Reactivity Index in 236 healthy adults aged 43–80 years (*M* = 60.30, *SD* = 6.88, 76% female).

**Results:**

Multiple linear regression analyses revealed that age only had a significant, medium, negative association with cognitive (*B* = −.08, *p* < .001) and affective (*B* = −.05, *p <* .001) ToM and was not significantly associated with emotion perception or emotional empathy.

**Conclusions:**

These findings enhance our understanding of normal social cognitive aging in later life, which can inform decisions around adding social cognitive measures into existing neuropsychological diagnostic tools for psychiatric, neurological, and developmental disorders.

##  

Social cognition is an umbrella term for cognitive abilities that involve comprehending stimuli related to individuals and their interactions (Happé et al., 2017). Although there is no consensus on what abilities fall under this umbrella category, we will define social cognition as the abilities of emotion perception, affective theory of mind (ToM), cognitive ToM, and emotional empathy. Emotion perception involves identifying universally recognizable emotions (i.e., happy, sad, anger, surprise, disgust, and fear) through verbal, bodily, and facial cues (Baron-Cohen et al., 2009; Mitchell & Phillips, 2015). A closely related but distinct ability is affective ToM, which involves inferring the affective state, emotion, or feeling of others (Duval et al., 2011). Unlike emotion perception, affective ToM is context and culture dependent, requiring more complex decision-making and reasoning abilities (Baron-Cohen et al., 2009; Mitchell & Phillips, 2015). This distinction has been supported by neurophysiological and behavioral evidence (Mitchell & Phillips, 2015). Cognitive ToM, in contrast, involves inferring the beliefs, thoughts, or intentions of others and can be further categorized by complexity into first-order inferences (i.e., adopting a single person’s perspective) and second-order inferences (i.e., simultaneously adopting the perspective of two individuals) (Duval et al., 2011). Finally, emotional empathy refers to the emotional response to the affective state of others (Baron-Cohen & Wheelwright, 2004). Although emotional empathy is primarily considered an emotional process, it is often included within the category of social cognition due to its significant overlap with affective ToM (Schurz et al., 2021).

Healthy social functioning in later life is closely linked to healthy aging and longevity (Stietz et al., 2021). A meta-analysis by Holt-Lunstad and coworkers (2010) identified that social relationships play a vital role in healthy aging, as poor social relationships were a significant risk factor for mortality. This risk was determined to be comparable with well-known factors such as smoking and alcohol consumption, even surpassing other risk factors such as physical inactivity. Furthermore, reduced social contact has been shown to increase the risk of general cognitive decline (Biddle et al., 2019; Kotwal et al., 2016; Lövdén et al., 2005). Emotional empathy is a fundamental prerequisite for higher social functioning (Bailey et al., 2008) and is associated with more meaningful social interactions (Guhn et al., 2020). ToM, when assessed using measures that combine the cognitive and affective subcomponents, has been found to have significant associations with social engagement among friends (Lecce et al., 2017) and family (Grainger et al., 2023). Therefore, investigating changes in social cognitive abilities in healthy adults can provide valuable insights into promoting healthy social functioning and overall wellbeing in later life.

Considerable research has examined performance differences between younger and older adults on emotion perception, cognitive ToM, affective ToM, and emotional empathy (Hayes et al., 2020; Henry et al., 2013; Jarvis et al., 2024; Ruffman et al., 2008). Meta-analytic findings indicate that compared to younger adults, older adults exhibit poorer performance in emotion perception (Hayes et al., 2020; Ruffman et al., 2008) and cognitive and affective ToM (Henry et al., 2013) and higher emotional empathy performance (Jarvis et al., 2024). Specifically, Ruffman and coworkers’ (2008) meta-analysis discovered varying age effect estimates for identifying neutral, angry, sad, disgusted, fearful, happy, and surprised expressions. Moderate effect sizes were reported for the identification of angry, sad, and fearful facial expressions (*r* = .34, .34, and .27, respectively), whereas small effect sizes were found for surprised and happy expressions (*r* = .07 and .08). Notably, age differences in the identification of disgusted facial expressions did not reach statistical significance. In the updated Hayes and colleagues (2020) meta-analysis, they found a very similar pattern of results when the effect of age was considered across all emotion perception measures. However, when the differential effects of video versus static image stimuli were considered, differential age effects for individual emotions were only found in measures employing static images. Video-based emotion perception measures found moderate age effects across all emotions, including disgust (Hayes et al., 2020). Concerning cognitive and affective ToM, Henry and coworkers’ (2013) meta-analysis reported large effect estimates for age-related reductions in cognitive ToM performance (*r* = −.51) and a moderate reduction in affective ToM (*r* = −.45), although they did not investigate performance differences for first-order and second-order cognitive ToM. This represents a significant gap in current knowledge, as research suggests that age-related impairments are more pronounced for second-order inferences than first-order inferences (Duval et al., 2011). Lastly, regarding emotional empathy, Jarvis and coworkers’ (2024) meta-analysis reported only a small effect size (Hedges’ *g* = 0.10), and the confidence interval of this effect estimate contained zero, leading to uncertainty regarding confidence in the conclusion that older adults have better performance.

Limited research has examined social cognitive performance across later life (i.e., adults in their 50s through to 90s). Given that the steepest declines in general cognitive functioning typically occur after 65 to 70 years of age (van der Willik et al., 2021), this is a significant gap in our current understanding of normal social cognitive functioning. Of the few studies that have investigated social cognitive performance throughout later life, the majority have only examined one or two domains rather than all four domains in the same sample (i.e., emotion perception, affective ToM, cognitive ToM, and emotional empathy).

For instance, Raimo and coworkers (2022) found reduced performance in older adults when investigating cognitive and affective ToM with medium effect sizes, but they did not consider the influence of both first- and second-order cognitive ToM. Gourlay and coworkers (2021) examined emotion perception in conjunction with cognitive ToM and identified reduced ability in older adults with large effect sizes (Gourlay et al., 2021). Maddaluno and coworkers (2022) reported a small positive association between age and emotional empathy and a medium negative association with affective ToM. McDonald and coworkers (2018) investigated multiple social cognitive domains, including emotion perception (without considering the influence of individual emotion identification), affective ToM, and first- and second-order cognitive ToM. They identified reduced ability across all domains but had a limited representation of adults over the age of 75. Therefore, to the best of our knowledge, no study has comprehensively examined all four social cognitive domains throughout the later lifespan within a single large sample, accounting for individual emotion identification and first- and second-order cognitive ToM. This approach allows for a more comprehensive understanding of the association between age and social cognition compared to previous studies, which have focused on single or limited domains, enabling age associations to be compared across social cognitive domains.

When investigating age-related trajectories of social cognition, it is important to consider four key factors: sex, years of education, negative mood status (i.e., depression and anxiety symptoms), and fluid intelligence quotient (IQ). Sex differences have been identified in all four social cognitive domains: women tend to score higher than men on tasks of emotion perception (Demenescu et al., 2014), affective ToM (Greenberg et al., 2022), cognitive ToM (Adenzato et al., 2017), and emotional empathy (Sommerlad et al., 2021). Similarly, higher levels of education have been associated with better performance in each domain: emotion perception (Demenescu et al., 2014), affective ToM (Stutesman & Frye, 2022), cognitive ToM (Li et al., 2013), and emotional empathy (Yaghoubi Jami et al., 2021). Negative mood status, including depression and anxiety symptoms, have been documented to affect social cognitive abilities. Depressive symptoms have been associated with reduced performance in emotion perception, affective ToM, cognitive ToM, and emotional empathy tasks (Bora & Berk, 2016; Zhang et al., 2021). Anxiety symptoms have shown a negative association with cognitive ToM (Braley et al., 2022) and a positive association with emotional empathy (Grainger et al., 2023). Fluid IQ e has been shown to be positively associated with emotion perception and cognitive and affective ToM performance (Baker et al., 2014; Di Tella et al., 2020; Kong et al., 2022). Given that fluid IQ declines with age (Bugg et al., 2006; Martin et al., 2021), to ensure that any age effects that may be uncovered reflect a change in the social cognitive process rather than fluid IQ, it is important to control for this in analyses.

The present study investigated the association between age and performance on four social cognitive measures within a sample of healthy midlife to older adults aged 43–80 years. To address existing gaps in the literature, this study will examine age-related cross-sectional associations for neutral and each of the six universal emotions (i.e., happiness, fear, disgust, anger, sadness, and surprise). Additionally, first-order cognitive ToM, second-order cognitive ToM, affective ToM, and emotional empathy will be examined. While controlling for the potential influence of sex, years of education, fluid IQ, and negative mood status on social cognitive performance, it was hypothesized that the perception of happy, fearful, angry, sad, surprised, and neutral facial expressions would be negatively associated with age, along with first-order cognitive, second-order cognitive, and affective ToM. The perception of disgusted facial expressions was hypothesized not to change significantly with age. Emotional empathy was proposed to show a positive association with age.

## METHOD

### Procedure and Design

A cross-sectional quantitative observational design was used. Informed consent was obtained from all participants before completing neuropsychological testing at the University of Queensland Centre for Clinical Research or the University of Queensland Neuropsychology Research Centre. The neuropsychological assessments were administered in the same order for all participants, and this was completed either before or after neuroimaging and blood sample protocols (detailed in Lupton and coworkers 2021). Demographic data, including participant age, sex, and years of education, were collected during neuropsychological assessments, which typically lasted 2 hr.

### Transparency and Openness

Due to privacy, confidentiality, and constraints imposed by the local Human Research Ethics Committee, data will be made available only upon request to the Prospective Imaging Study of Ageing team at QIMR Berghofer Medical Research Institute. Analyses were performed in R version 4.2.2 (R Core Team, 2021) using packages “corrplot” (Wei & Simko, 2017), “car” (Fox & Weisberg, 2019), “lmtest” (Zeileis & Hothorn, 2002), and “sandwich” (Zeileis et al., 2020). The analysis code is available publicly on GitHub (Jarvis, 2023). This study’s design and its analysis were not preregistered.

### Participants

The current study utilized data from the Prospective Imaging Study of Ageing (Lupton et al., 2021), which aimed to characterize and phenotype healthy adult Australians at high risk of Alzheimer’s disease. Power analyses using the R package “pwrss” (Bulus, 2023) determined that a minimum sample of 163 participants was required to detect an *R*^2^ of .10, power of .80, and alpha of .017 in multiple regression analyses with six predictors entered in the model. This *R*^2^ was determined based on the ANCOVA results reported by Gourlay and coworkers (2021), which examined cognitive ToM (*F* (2,116) *=* 20.09, *p* < .001) and emotion perception (*F* (2,115) *=* 13.46, *p* < .001) performance across age groups with years of education entered as the covariate analyses; the lower estimate was used and converted into *R*^2^ using the formula *R*^2^ = *F*/(*F* + *df*). The final sample comprised 236 adults aged 43–80 years (*M* = 60.30, *SD* = 6.88, 76% female) and had a mean of 13.5 years (*SD* = 2.88) of education. Participants were recruited from the metropolitan area of Brisbane, Australia, and were primarily Caucasian from a Western cultural background who spoke English as their primary language. All data were collected between 2017 and 2020. Martin and colleagues (2022) reported a subset of these participants in their examination of executive functioning and summary social cognition measures (social inference and total emotion perception).

Relevant exclusion for the current study were (1) diagnosis of a neurological disorder, (2) history of neurosurgery, (3) any significant medical condition that may confound neuropsychological testing, (4) current alcohol or substance abuse, (5) history of severe psychiatric illness, or (6) current psychiatric symptoms that may confound neuropsychological testing. This study was approved by the Human Research Ethics Committees of QIMR Berghofer Medical Research Institute and The University of Queensland. All participants were checked to ensure their cognitive profile did not meet the diagnostic criteria for mild cognitive impairment or Alzheimer’s disease, as detailed in Lupton and coworkers (2021).

### Measures

#### Emotion perception

The Mini-SEA Facial Emotion Recognition Test (Bertoux et al., 2014) was used to measure emotion perception. It comprises 35 images of facial expressions selected from Ekman pictures (Ekman & Friesen, 1975) and requires participants to identify which of seven possible emotions (fear, sadness, disgust, surprise, anger, happiness, and neutral) is expressed in the image. The Mini-SEA Facial Emotion Recognition Test demonstrates good reliability, with a reported test–retest correlation of *r* = .634 (Mulet-Perreault et al., 2024). A correct score for each individual emotion (ranging from 0 to 5 each) and total emotion perception across all emotions (ranging from 0 to 35) was examined, with higher scores representing better emotion perception.

#### Cognitive and affective theory of mind

The Shortened Awareness of Social Inference Test—Short Form (TASIT-S) Part 2 Social Inference Minimal (Honan et al., 2016) was used to measure cognitive and affective ToM. Rasch analysis indicates that this measure has high item reliability (Honan et al., 2016). Participants were required to watch nine short video vignettes of social interactions between trained male and female actors. After viewing each scene, participants answered three probe questions targeting their understanding of what the actors were doing, thinking, and feeling. These “think” questions measured first-order cognitive ToM, “do” questions measured second-order cognitive ToM, and “feel” questions measured affective ToM. Participants answered Yes, No, or Don’t Know for each probe question. Additionally, participants answered a fourth control probe question, “say,” to test their story comprehension; these responses were not directly examined in the current study. TASIT-S responses were examined as a total score for each type of probe question (i.e., do, think, and feel, ranging from 0 to 9 each, with higher scores representing better ToM performance).

#### Emotional empathy

The Interpersonal Reactivity Index (IRI), Empathic Concern, and Personal Distress subscales (Davis, 1980) measured emotional empathy. Each subscale includes seven items evaluated on a 5-point Likert scale ranging from 0 (does not describe me well) to 4 (describes me very well). The Empathic Concern subscale measures the tendency to experience warmth, compassion, and concern for others (e.g., “I often have tender, concerned feelings for people less fortunate than me”). The Personal Distress subscale measures personal unease and discomfort experienced in reaction to others’ emotions (e.g., “Being in a tense emotional situation scares me”). The IRI has acceptable internal consistency, with Cronbach’s α ranging from .67 to .87 (Hawk et al., 2012). A total score for each subscale (ranging from 0 to 28 each) and for the two subscales combined (ranging from 0 to 56) was examined, with higher scores representing greater empathic concern or personal distress.

##### Current mood symptoms

The Hospital Anxiety and Depression Scale (HADS) (Zigmond & Snaith, 1983) measured anxiety and depression symptoms and consisted of 14 items, 7 items each for the anxiety (HADS-A) and depression (HADS-D) subscales. This measure has strong internal consistency, with Cronbach’s α ranging from .80 to .93 (Herrmann, 1997). Each item is measured on a scale from 0 to 3. A total score for each subscale (ranging from 0 to 21) was examined, with higher scores representing greater anxiety and depression severity.

##### Fluid intelligence

Participants’ fluid IQ was examined using the Matrix Reasoning subtest of the Wechsler Abbreviated Scale of Intelligence (WASI-II) (Wechsler, 2011), which entails the completion of visual matrices consisting of patterns or sequences of shapes, symbols, or designs. WASI-II has excellent internal consistency, with Cronbach’s α ranging from .94 to .97 (Wechsler, 2011). Participants were required to accurately identify and place the missing piece that ensures logical completion of the matrix. Possible scores could range from 0 to 30, with higher scores representing higher fluid IQ.

### Statistical Approach

The analyses examined 15 outcome variables categorized into three social cognition domains: emotion perception, ToM, and emotional empathy. Emotion perception comprised eight variables: happiness, fear, disgust, anger, surprise, sad, neutral, and total emotion perception. ToM comprised four variables: “think” (first-order cognitive ToM), “do” (second-order cognitive ToM), total cognitive ToM (the sum of “do” and “think” scores), and “feel” (affective ToM). Emotional empathy comprised three outcome variables: empathic concern, personal distress, and total emotional empathy.

Correlations between each of the 15 outcome variables were identified using Spearman’s rank correlation coefficient. Weak, moderate, and strong effects were classified as .20, .40, and .60, respectively.

A total of 15 multiple linear regressions for each outcome variable were run, with age entered as the dependent variable and the following entered as co-variables: sex, years of education, anxiety, depression, and fluid IQ. These were entered as co-variables in the regression analyses only, as the aim of this study was to merely examine the association between age and social cognition while controlling for the effects of these other variables known to influence performance. The goal of this study was not to examine the specific influence of these variables on social cognitive performance. Spearman’s rank correlation coefficients for each control variable are reported in Supplementary materials. Sex was coded as 1 = male or 2 = female.

Each multiple linear regression was checked for the following assumptions: normality of residuals, linearity, and homoscedasticity. The assumption of linearity was examined through the visual examination of residuals versus fitted plots, which confirmed that the data were linear. The normality of residuals was assessed via the visual examination of QQ plots and Shapiro–Wilk and homoscedasticity via scale-location plots and the Breusch–Pagan test. Most models did not display normally distributed residuals, and many were heteroscedastic, so robust regressions were run using robust covariance matrix estimation from the r package “sandwich” (Zeileis et al., 2020). Specifically, we used heteroscedasticity-consistent estimators, which adjust the standard errors of the regression coefficients to account for this heteroscedasticity. To correct for multiple comparisons, alpha was divided by the number of social cognitive domains examined (i.e., three), resulting in an α of .017 for each regression. While hierarchical linear regressions were considered, due to the data not meeting the assumptions of linearity and homoscedasticity, these could not be run. Instead, minimally adjusted regression models with only age and sex included were also run for each outcome variable. This allows for any changes in age associations after the inclusion of all control variables in the model to be observed. As these regressions did not meaningfully differ from the full models regarding direction, effect size, or statistical significance, these results are only reported in Supplementary materials. Small, medium, and large effect sizes were quantified as β <.02, ≥.2, and ≥.5, respectively (Fey et al., 2023).

## RESULTS

**Fig. 1:**
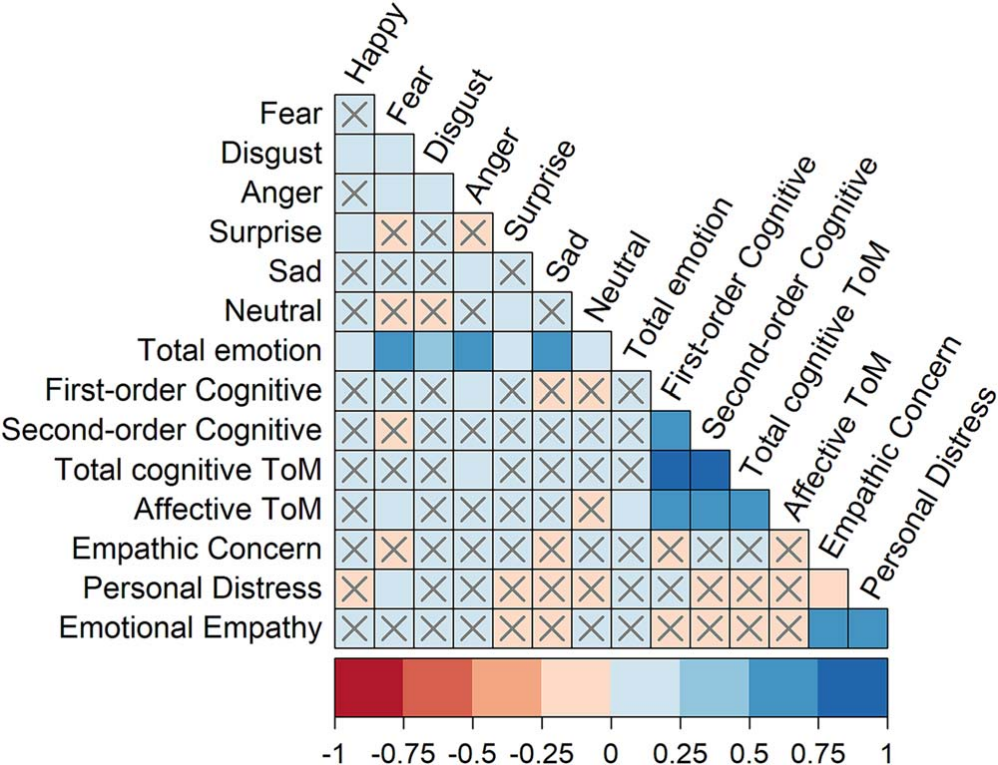
Spearman’s rank correlation coefficients between each of the outcome variables. *Note. The x-axis* indicates Spearman’s *ρ* value. × represents non-significance (*p* > .050), while the absence of × represents significance (*p* < .050).

Table 1 displays descriptive statistics for all variables, and Fig. 1 shows Spearman’s rank correlation coefficients between key variables; for specific values of correlation coefficients, see Supplementary Materials. ToM variables were moderate to strongly positively correlated with one another, while the different emotions tended to be weakly correlated with one another or were not statistically significant. The two emotional empathy variables of empathic concern and personal distress were weakly negatively correlated with one another. Correlations between social cognitive subdomain variables (i.e., emotion perception, theory of mind, and emotional empathy) tended to be weak and not statistically significant.

**Table 1:** Descriptive statistics for all variables

Variables	*N* (Range)	*M* (*SD*)
Age (total sample)	234 (43–80)	60.30 (6.88)
40–49 years	21	47.10 (2.05)
50–59 years	80	55.70 (2.54)
60–69 years	113	64.00 (2.64)
70–80 years	20	71.80 (2.63)
Education	234 (8–22)	13.50 (2.88)
Anxiety (max score 21)	232 (0–18)	5.44 (3.41)
Depression (max score 21)	232 (0–15)	2.56 (2.60)
Fluid IQ (max score 31)	234 (6–28)	18.44 (3.79)
Happy (max score 5)	218 (2–5)	4.94 (0.31)
Fear (max score 5)	218 (0–5)	2.20 (1.36)
Disgust (max score 5)	218 (1–5)	4.17 (0.93)
Anger (max score 5)	218 (0–5)	3.78 (0.99)
Surprise (max score 5)	218 (2–5)	4.62 (0.66)
Sad (max score 5)	218 (0–5)	3.44 (1.43)
Neutral (max score 5)	218 (3–5)	4.81 (0.44)
Total emotion perception (max score 35)	218 (17–35)	27.95 (3.03)
First-order cognitive (max score 9)	193 (4–9)	7.58 (1.23)
Second-order cognitive (max score 9)	193 (4–9)	7.73 (1.18)
Total cognitive ToM (max score 18)	193 (9–18)	15.31 (2.19)
Affective ToM (max score 9)	193 (5–9)	7.76 (1.12)
Empathic concern (max score 28)	184 (7–28)	18.14 (4.78)
Personal distress (max score 28)	185 (1–28)	10.37 (4.55)
Emotional empathy total (max score 56)	183 (12–52)	28.51 (6.27)

ToM, theory of mind.

**Fig. 2:**
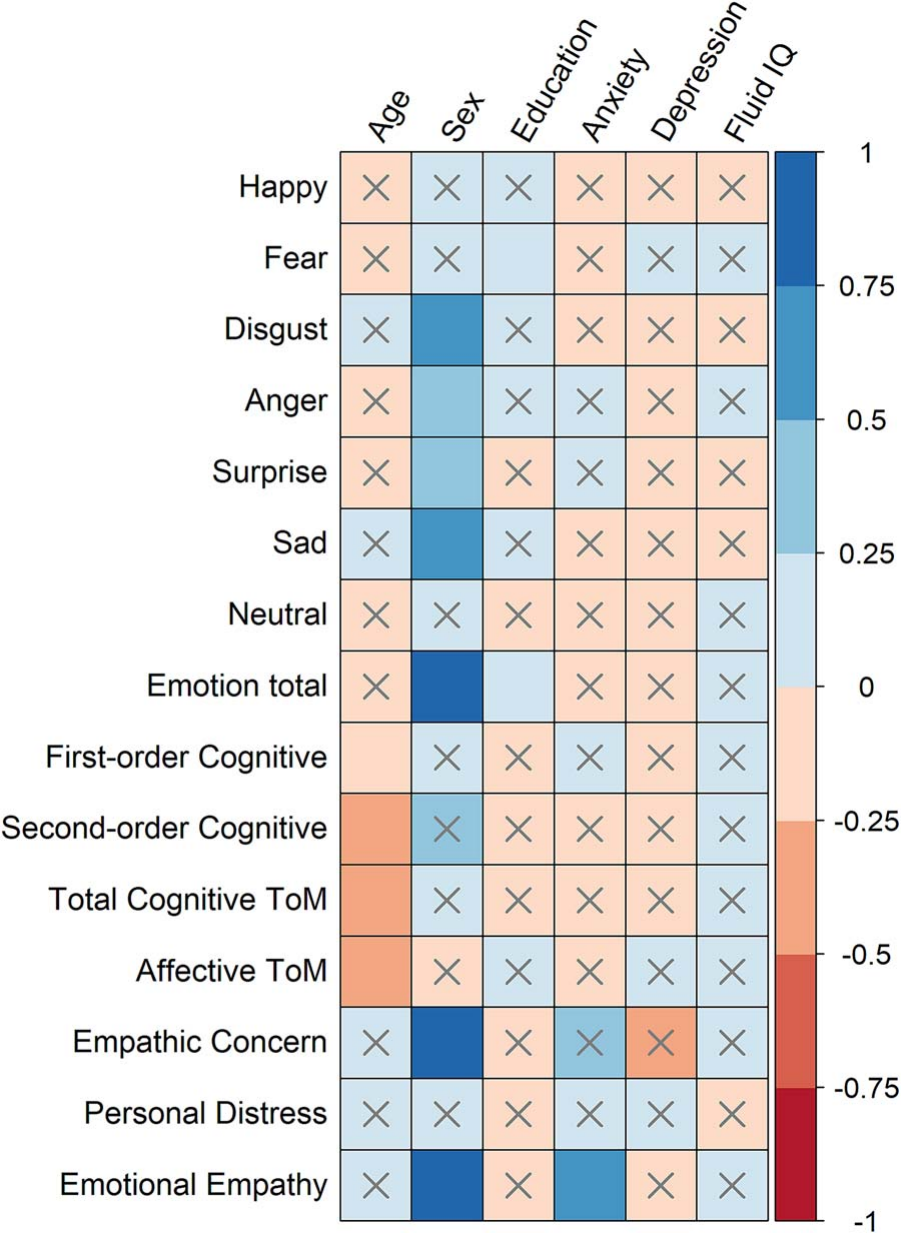
Fifteen multiple linear robust regression models accounting for seven controls, displaying standardized β values and if the effect reached statistical significance (*p* < .017). *Note.* The y-axis indicates β value. × represents non-significance (*p* > .017), while the absence of × represents significance (*p* < .017).

**Fig. 3:**
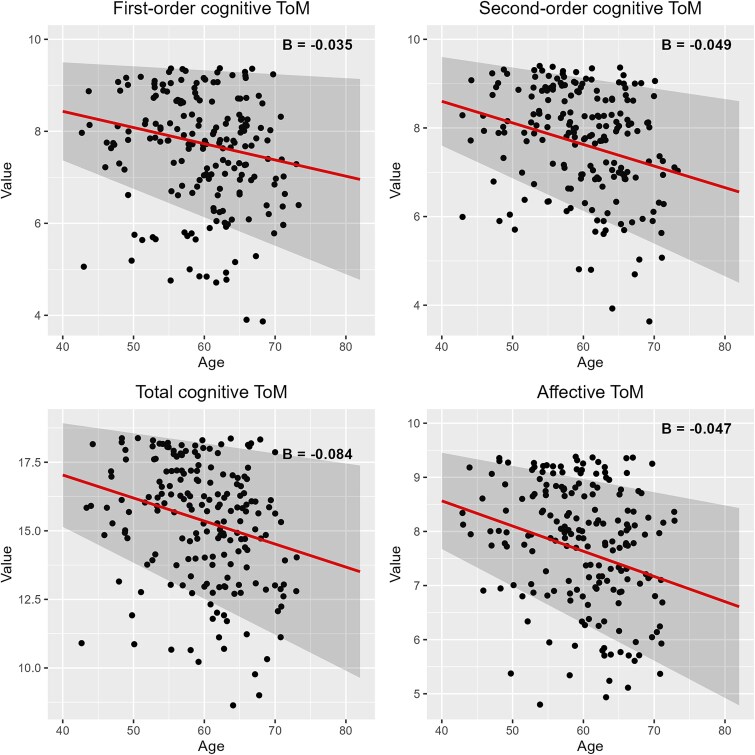
Visual comparison of unstandardized *B* values for age across four significant social cognition outcome variables. *Note.* Slope of the line is equal to *B* (the unstandardized coefficient)*.* Intercept is equal to the intercept of robust multiple regression model. All *B* were significant (*p* < .017), indicating that age was a significant predictor of the domain’s total score. Shaded areas indicate 95% confidence interval.

The results of the 15 multiple linear regressions can be viewed in Fig. 2 (see also Supplementary Materials). Age was negatively and moderately associated with first-order cognitive ToM (β = −.20, *p* = .010), second-order cognitive ToM (β = −.28, *p* < .001), total cognitive ToM (β = −.26, *p <* .001), and affective ToM (β = −.29, *p* < .001), but was not a significant predictor for any of the emotion perception or emotional empathy outcome variables. The association of age with each of the four ToM variables are displayed in Fig. 3, while all non-significant associations for the remaining social cognitive variables are shown in Supplementary Materials.

Being female sex was a large predictor of disgust (β = .59, *p* = .001), anger (β = .45, *p* = .012), surprise (β = .45, *p* = .009), sad (β = .53, *p* = .002), total emotion perception (β = .79, *p <* .001), empathic concern (β = .49, *p* = .006), and emotional empathy total (β = .52, *p* = .002). Education was positively and moderately associated with fearful emotions (β = .21, *p* = .004) and total emotion perception (β = .19, *p* = .006), while anxiety was positively and moderately associated with emotional empathy total (β = .30, *p* = .002). Depression and fluid IQ were not significant predictors of any of the 15 total outcome variables.

As fluid IQ was not a significant predictor of social cognitive performance in the current sample, to ensure that the sample displayed the typical declines known to occur in later life (Salthouse, 2004), a simple linear regression between fluid IQ and age was run, demonstrating a small, negative association with age but it did not reach conventional significance levels (β = −.12, *p* = .080).

## DISCUSSION

In a sample of >200 mid- to late-life healthy adults, using a comprehensive social cognition battery, we observed age-related declines in ToM despite a lack of age-related change in emotion perception and emotional empathy. Consistent with our initial hypothesis, findings demonstrated an age-related decline in ToM performance for first-order cognitive, second-order cognitive, and affective ToM, with medium effect sizes identified. Contrary to our expectations, age was not significantly associated with emotional empathy or emotion perception in either the full or minimally adjusted models. It appears as though social cognitive domains display different patterns of age-related change, just like non-social cognitive domains (Salthouse, 2004).

The negative association between age and affective, first-order cognitive, and second-order cognitive ToM supports previous findings (Charlton et al., 2009; Johansson Nolaker et al., 2018; McDonald et al., 2018). These results have important implications for future research, given that, to date, limited research examining all four subdomains in later life exists. Additionally, this negative association suggests that these abilities may exhibit similar declines observed in general cognitive functions such as memory and processing speed (Salthouse, 2004) and in executive functions such as initiation, inhibition, and strategy use (Gibson et al., 2019). Given the established importance of ToM for social engagement (Grainger et al., 2023; Lecce et al., 2017), which, in turn, contributes to healthy aging and longevity (Stietz et al., 2021), our findings hold significant implications for the investigation of intervention strategies aimed at mitigating these ToM deficits in older adults. A promising avenue for intervention lies in conversation-based training, which has demonstrated efficacy in improving ToM skills among older adults aged ≥65 years (Cavallini et al., 2021; Lecce et al., 2015).

The use of a highly ecologically valid measure of ToM performance through video vignettes of interactions with trained actors was both a strength and weakness of the current study. The high ecological validity was a strength due to the enhanced generalizability of our findings to real-world social interactions, thereby increasing the practical relevance of our results. However, as the video vignettes were developed for Western, English-speaking populations only, this is also a considerable weakness of the current study and highlights the need for the development of ecologically valid ToM measures for diverse cultural backgrounds. Relatedly, cultural and linguistic differences have been demonstrated to influence ToM performance, which the current study did not consider. A study investigating ToM performance across 12 countries found that between 21% and 25% of the variance was explained by country of origin (Quesque et al., 2022). Similarly, in a cross-sectional examination of whether age effects in ToM differ based on culture, Yong and coworkers (2022) identified that deficits were more amplified in Malaysian compared with U.K. samples. Regarding linguistics, meta-analyses have uncovered associations in children between ToM performance and language ability (Milligan et al., 2007) and bilingualism (Schroeder, 2018). However, few studies have examined whether this association exists in adults, especially older adults (Feng et al., 2023; Miller, 2022). One study that examined the influence of language on cognitive ToM performance in young, middle, and older adults did not find a significant association (Bernstein et al., 2011). A study on ToM performance differences in men aged 74 years found that there was a trend for bilinguals to outperform monolinguals, although the results were not statistically significant (Cox et al., 2016). Future research should examine whether controlling for culture and language modifies the negative relationship between age and ToM performance.

In contrast to our expectations, our findings revealed no significant association between age and emotion perception, including the identification of individual emotions (i.e., happy, sad, angry, fearful, disgusted, surprised, or neutral expressions). This contradicts previous research that reported reduced performance in older adults compared to younger adults (Hayes et al., 2020; Ruffman et al., 2009) and negative associations across the later life span for at least some emotions (Horning et al., 2012; Moraitou et al., 2013). One potential explanation of our results could be that older adults exhibit reduced emotion perception ability compared to younger adults, although these declines do not continue to change significantly throughout later life. Our findings may indicate a leveling off or stabilization of emotion perceptual abilities beyond a certain age. It is also important to acknowledge the limitations of our emotion perception measure examined, mini-SEA, which employed static pictures of facial expressions and had lower ecological validity. In contrast, Moraitou and coworkers’ (2013) study utilized video vignettes of trained actors, ensuring higher ecological validity. It is possible that measures with greater ecological validity are more sensitive to detecting age effects.

Our study did not find a significant association between age and emotional empathy performance, which partly aligns with Jarvis and coworkers’ (2024) meta-analytic subgroup analyses focusing on whether different emotional empathy measures exhibit different age-related associations. In these analyses, Davis’ (1980) IRI Empathic Concern subscale did not significantly differ between younger and older adults, as in the current study. However, a negative association was observed for the IRI Personal Distress subscale, diverging from the current results. It has been suggested that the IRI indexes processes broader than emotional empathy, including sympathy, emotional self-control, and affective ToM (Baron-Cohen & Wheelwright, 2004; Chrysikou & Thompson, 2016; Jolliffe & Farrington, 2006). The IRI’s broader measurement of related processes results in different patterns of age-related changes to emotional empathy–specific measures (Jarvis et al., 2024). Despite these limitations with the IRI, our results still support those by Grainger and coworkers (2023) who used a more ecologically valid assessment method.

To the best of our knowledge, this was the first study to comprehensively examine social cognitive performance across later life within a single large sample. Notably, it takes into consideration the potentially confounding effects of sex, years of education, fluid IQ, and mood status. This contribution significantly enhances existing literature, enabling a deeper understanding of age-related trajectories of social cognitive performance throughout later life than previously undertaken. Additionally, this study enriches our understanding of cognitive aging theory. While extensive knowledge exists of how other cognitive abilities, including memory, processing speed, reasoning, and executive functioning, change with advancing age (Martin et al., 2021; Salthouse, 2019; van der Willik et al., 2021), this is not clear for social cognitive abilities. Demonstrating the importance of informing cognitive aging theory related to social cognitive abilities is research indicating that social cognition is associated with better social functioning in nursing home residents (Washburn et al., 2003). Additionally, meta-analytic findings indicate that 30 different psychiatric, neurological, and developmental disorders that exhibit significant impairments in social functioning also demonstrate marked social cognitive deficits (Cotter et al., 2018). Adding social cognitive measures to existing neuropsychological screening tools used to diagnose these disorders has important clinical applications, but first, we must enrich our understanding of normal social cognitive aging.

Three notable limitations of the current study, which limit our understanding of typical social cognitive functioning, need to be acknowledged. The first is that the mean age of the sample skewed younger at 60 years of age, and there were no adults over the age of 80 years. Therefore, we still do not clearly understand normal social cognitive functioning in the oldest of the old. The second significant limitation is that our sample consisted primarily of WEIRD (Western, Educated, Industrialized, Rich, and Democratic) adults who tend to have higher education levels than the broader population. Therefore, more research must be conducted utilizing culturally diverse populations and older samples that are more representative of modern life expectancies. Relatedly, such research must also carefully consider the measures used to examine social cognitive performance to ensure that they are not limited by the generalizability issues that are present in culture- and language-based tools such as that used in the current study, TASIT. Lastly, given this study used a cross-sectional design, no causative conclusions about the association between age and social cognition can be drawn. Longitudinal research will be required to determine whether it is age that is driving these results or other confounding variables.

In summary, the current study revealed that age was only significantly negatively associated with cognitive and affective ToM performance, while no significant age-related associations were observed for emotion perception or emotional empathy. These results suggest that only cognitive and affective ToM abilities may continue to reduce as individuals age. Importantly, this study contributes to the existing literature by examining a comprehensive measure of social cognition while controlling for factors that previous research has indicated may influence age associations. An important implication of enhancing our understanding of normal social cognitive aging is that it could help improve the clinical utility of social cognitive measures in existing neuropsychological screening and diagnostic tools for disorders that exhibit significant social cognitive deficits.

## Supplementary Material

Supplementary_Materials_CLEAN_acaf022
