# Supplementary material for: Cross-Sectional Trajectories of Social Cognition in Later Life: Exploring Emotion Perception, Theory of Mind, and Emotional Empathy
Source: Arch Clin Neuropsychol. 2025 Mar 9;40(6):1091–100. doi: 10.1093/arclin/acaf022 (PMC12378355; doi:10.1093/arclin/acaf022)
Supplement: Supplementary_Materials_CLEAN_acaf022 [file supplementary_materials_clean_acaf022.docx]

**Supplementary Table 1.**

*Spearman’s Rank Correlation Coefficients for each outcome variable.*

*Note. p* values are in brackets.

|  | M (SD) | 1 | 2 | 3 | 4 | 5 | 6 | 7 | 8 | 9 | 10 | 11 | 12 | 13 | 14 |
| --- | --- | --- | --- | --- | --- | --- | --- | --- | --- | --- | --- | --- | --- | --- | --- |
| **Emotion perception** |  |  |  |  |  |  |  |  |  |  |  |  |  |  |  |
| 1. Happy | 4.94 (0.31) |  |  |  |  |  |  |  |  |  |  |  |  |  |  |
| 2. Fear | 2.20 (1.36) | 0.02 |  |  |  |  |  |  |  |  |  |  |  |  |  |
| 3. Disgust | 4.17 (0.93) | **0.16** | **0.15** |  |  |  |  |  |  |  |  |  |  |  |  |
| 4. Anger | 3.78 (0.99) | 0.09 | **0.14** | **0.19** |  |  |  |  |  |  |  |  |  |  |  |
| 5. Surprise | 4.62 (0.66) | **0.22** | -0.03 | 0.05 | -0.02 |  |  |  |  |  |  |  |  |  |  |
| 6. Sad | 3.44 (1.43) | 0.00 | 0.07 | 0.02 | **0.15** | 0.00 |  |  |  |  |  |  |  |  |  |
| 7. Neutral | 4.81 (0.44) | 0.06 | -0.03 | -0.07 | 0.04 | **0.14** | 0.07 |  |  |  |  |  |  |  |  |
| 8. Total Emotion Perception | 27.95 (3.03) | **0.19** | **0.61** | **0.46** | **0.52** | **0.20** | **0.58** | **0.16** |  |  |  |  |  |  |  |
| **ToM** |  |  |  |  |  |  |  |  |  |  |  |  |  |  |  |
| 9. First-order cognitive | 7.58 (1.23) | 0.13 | 0.07 | 0.06 | **0.15** | 0.13 | -0.02 | -0.01 | 0.14 |  |  |  |  |  |  |
| 10. Second-order cognitive | 7.73 (1.18) | 0.11 | -0.01 | 0.02 | 0.14 | 0.04 | 0.04 | 0.03 | 0.11 | **0.61** |  |  |  |  |  |
| 11. Total Cognitive ToM | 15.31 (2.19) | 0.12 | 0.04 | 0.04 | **0.16** | 0.09 | 0.01 | 0.02 | 0.14 | **0.90** | **0.89** |  |  |  |  |
| 12. Affective ToM | 7.76 (1.12) | 0.06 | **0.16** | 0.01 | 0.14 | 0.03 | 0.06 | -0.06 | **0.16** | **0.65** | **0.59** | **0.69** |  |  |  |
| **Emotional empathy** |  |  |  |  |  |  |  |  |  |  |  |  |  |  |  |
| 13. Empathic concern | 18.14 (4.78) | 0.02 | -0.06 | 0.06 | 0.05 | 0.04 | -0.03 | 0.12 | 0.01 | -0.01 | 0.02 | 0.01 | -0.11 |  |  |
| 14. Personal distress | 10.37 (4.55) | -0.02 | **0.16** | 0.01 | 0.02 | -0.04 | -0.00 | -0.08 | 0.08 | 0.01 | -0.10 | -0.05 | -0.03 | **-0.19** |  |
| 15. Total emotional empathy | 28.51 (6.27) | 0.01 | 0.06 | 0.07 | 0.06 | -0.02 | -0.00 | 0.04 | 0.08 | -0.02 | -0.04 | -0.03 | -0.14 | **0.62** | **0.60** |

*Note.* Bolded p-values are significant (*p*<0.05).

**Supplementary Table 2.**

*Summary statistics for each of the 15 multiple linear regression models with robust standard errors, including each of the six predictors.*

| Variable | *R^2^* | *B* | *β* | *95%CI* | | *p* |
| --- | --- | --- | --- | --- | --- | --- |
| **Emotion Perception**  Happy | 0.01  0.01 |  |  |  |  |  |
| Age |  | -0.00 | -0.05 | -0.01 | 0.00 | 0.355 |
| Sex |  | 0.03 | 0.11 | -0.07 | 0.13 | 0.518 |
| Education |  | 0.00 | 0.01 | -0.02 | 0.02 | 0.909 |
| Anxiety |  | -0.00 | -0.02 | -0.02 | 0.02 | 0.872 |
| Depression |  | -0.00 | -0.03 | -0.03 | 0.02 | 0.745 |
| Fluid IQ |  | -0.01 | -0.10 | -0.02 | 0.00 | 0.120 |
| Minimally adjusted model 0.00 | | | | | | |
| Age |  | -0.00 | -0.03 | -0.01 | 0.00 | 0.481 |
| Sex |  | 0.03 | 0.08 | -0.07 | 0.12 | 0.590 |
| Fear | 0.07 |  |  |  |  |  |
| Age |  | -0.02 | -0.08 | -0.05 | 0.01 | 0.265 |
| Sex |  | 0.29 | 0.21 | -0.14 | 0.72 | 0.187 |
| Education |  | 0.10 | 0.21 | 0.03 | 0.16 | 0.004 |
| Anxiety |  | -0.02 | -0.04 | -0.09 | 0.06 | 0.678 |
| Depression |  | 0.03 | 0.05 | -0.05 | 0.11 | 0.504 |
| Fluid IQ |  | 0.03 | 0.08 | -0.02 | 0.08 | 0.221 |
| Minimally adjusted model 0.02 | | | | | | |
| Age |  | -0.02 | -0.11 | -0.05 | 0.00 | 0.096 |
| Sex |  | 0.25 | 0.18 | -0.18 | 0.68 | 0.255 |
| Disgust | 0.07 |  |  |  |  |  |
| Age |  | 0.01 | 0.10 | -0.01 | 0.03 | 0.197 |
| Sex |  | 0.55 | 0.59 | 0.21 | 0.88 | 0.001 |
| Education |  | 0.02 | 0.07 | -0.02 | 0.07 | 0.311 |
| Anxiety |  | -0.01 | -0.04 | -0.06 | 0.04 | 0.630 |
| Depression |  | -0.01 | -0.02 | -0.07 | 0.06 | 0.833 |
| Fluid IQ |  | -0.01 | -0.04 | -0.05 | 0.02 | 0.559 |
| Minimally adjusted model 0.06 | | | | | | |
| Age |  | 0.02 | 0.12 | -0.00 | 0.03 | 0.092 |
| Sex |  | 0.50 | 0.54 | 0.18 | 0.82 | 0.002 |
| Anger | 0.08 |  |  |  |  |  |
| Age |  | -0.01 | -0.08 | -0.03 | 0.01 | 0.252 |
| Sex |  | 0.45 | 0.45 | 0.10 | 0.79 | 0.012 |
| Education |  | 0.02 | 0.06 | -0.02 | 0.07 | 0.361 |
| Anxiety |  | 0.00 | 0.01 | -0.05 | 0.05 | 0.932 |
| Depression |  | -0.01 | -0.02 | -0.08 | 0.06 | 0.826 |
| Fluid IQ |  | 0.04 | 0.15 | 0.00 | 0.08 | 0.042 |
| Minimally adjusted model 0.05 | | | | | | |
| Age |  | -0.02 | -0.11 | -0.04 | 0.01 | 0.152 |
| Sex |  | 0.44 | 0.44 | 0.10 | 0.77 | 0.010 |
| Surprise | 0.06 |  |  |  |  |  |
| Age |  | -0.01 | -0.13 | -0.03 | 0.00 | 0.095 |
| Sex |  | 0.30 | 0.45 | 0.08 | 0.52 | 0.009 |
| Education |  | -0.01 | -0.03 | -0.04 | 0.02 | 0.654 |
| Anxiety |  | 0.00 | 0.01 | -0.03 | 0.04 | 0.886 |
| Depression |  | -0.02 | -0.08 | -0.06 | 0.02 | 0.339 |
| Fluid IQ |  | -0.01 | -0.04 | -0.03 | 0.02 | 0.529 |
| Minimally adjusted model 0.05 | | | | | | |
| Age |  | -0.01 | -0.11 | -0.03 | 0.00 | 0.142 |
| Sex |  | 0.29 | 0.44 | 0.07 | 0.51 | 0.009 |
| Sad | 0.07 |  |  |  |  |  |
| Age |  | 0.02 | 0.08 | -0.01 | 0.05 | 0.295 |
| Sex |  | 0.76 | 0.53 | 0.27 | 1.25 | 0.002 |
| Education |  | 0.06 | 0.12 | -0.00 | 0.12 | 0.064 |
| Anxiety |  | -0.03 | -0.06 | -0.10 | 0.05 | 0.483 |
| Depression |  | -0.00 | -0.01 | -0.10 | 0.09 | 0.927 |
| Fluid IQ |  | -0.04 | -0.10 | -0.09 | 0.02 | 0.179 |
| Minimally adjusted model 0.05 | | | | | | |
| Age |  | 0.02 | 0.09 | -0.01 | 0.05 | 0.203 |
| Sex |  | 0.68 | 0.48 | 0.21 | 1.15 | 0.005 |
| Neutral | 0.02 |  |  |  |  |  |
| Age |  | -0.00 | -0.01 | -0.01 | 0.01 | 0.900 |
| Sex |  | 0.01 | 0.03 | -0.14 | 0.16 | 0.868 |
| Education |  | -0.02 | -0.10 | -0.04 | 0.01 | 0.194 |
| Anxiety |  | -0.00 | -0.00 | -0.02 | 0.02 | 0.972 |
| Depression |  | -0.02 | -0.10 | -0.05 | 0.01 | 0.274 |
| Fluid IQ |  | 0.01 | 0.06 | -0.01 | 0.02 | 0.375 |
| Minimally adjusted model 0.00 | | | | | | |
| Age |  | 0.00 | 0.00 | -0.01 | 0.01 | 0.995 |
| Sex |  | 0.02 | 0.06 | -0.12 | 0.17 | 0.733 |
| Total emotion perception | 0.15 |  |  |  |  |  |
| Age |  | -0.01 | -0.03 | -0.07 | 0.05 | 0.644 |
| Sex |  | 2.40 | 0.79 | 1.47 | 3.32 | 0.000 |
| Education |  | 0.19 | 0.18 | 0.05 | 0.32 | 0.006 |
| Anxiety |  | -0.06 | -0.07 | -0.21 | 0.08 | 0.397 |
| Depression |  | -0.04 | -0.03 | -0.21 | 0.14 | 0.697 |
| Fluid IQ |  | 0.01 | 0.02 | -0.10 | 0.12 | 0.826 |
| Minimally adjusted model | 0.10 |  |  |  |  |  |
| Age |  | -0.01 | -0.03 | -0.07 | 0.04 | 0.613 |
| Sex |  | 2.20 | 0.73 | 1.31 | 3.10 | 0.00 |
| **Theory of Mind**  First-order cognitive | 0.05 |  |  |  |  |  |
| Age |  | -0.04 | -0.20 | -0.06 | -0.01 | 0.010 |
| Sex |  | 0.08 | 0.07 | -0.31 | 0.48 | 0.673 |
| Education |  | -0.03 | -0.07 | -0.09 | 0.03 | 0.375 |
| Anxiety |  | 0.00 | 0.01 | -0.06 | 0.07 | 0.920 |
| Depression |  | -0.03 | -0.06 | -0.13 | 0.07 | 0.550 |
| Fluid IQ |  | 0.02 | 0.07 | -0.03 | 0.07 | 0.392 |
| Minimally adjusted model | 0.04 |  |  |  |  |  |
| Age |  | -0.04 | -0.21 | -0.06 | -0.01 | 0.005 |
| Sex |  | 0.13 | 0.11 | -0.26 | 0.52 | 0.509 |
| Second-order cognitive | 0.11 |  |  |  |  |  |
| Age |  | -0.05 | -0.28 | -0.07 | -0.02 | 0.000 |
| Sex |  | 0.34 | 0.29 | -0.03 | 0.71 | 0.073 |
| Education |  | -0.06 | -0.14 | -0.11 | -0.00 | 0.045 |
| Anxiety |  | -0.02 | -0.06 | -0.08 | 0.04 | 0.515 |
| Depression |  | -0.02 | -0.05 | -0.10 | 0.06 | 0.588 |
| Fluid IQ |  | 0.01 | 0.03 | -0.04 | 0.05 | 0.733 |
| Minimally adjusted model 0.09 | | | | | | |
| Age |  | -0.04 | -0.26 | -0.07 | -0.02 | 0.001 |
| Sex |  | 0.35 | 0.30 | -0.01 | 0.71 | 0.054 |
| Total cognitive ToM | 0.09 |  |  |  |  |  |
| Age |  | -0.08 | -0.26 | -0.13 | -0.04 | 0.001 |
| Sex |  | 0.43 | 0.19 | -0.28 | 1.13 | 0.232 |
| Education |  | -0.09 | -0.11 | -0.19 | 0.02 | 0.120 |
| Anxiety |  | -0.02 | -0.03 | -0.13 | 0.10 | 0.776 |
| Depression |  | -0.05 | -0.06 | -0.22 | 0.12 | 0.538 |
| Fluid IQ |  | 0.03 | 0.05 | -0.05 | 0.11 | 0.479 |
| Minimally adjusted model 0.08 | | | | | | |
| Age |  | -0.08 | -0.26 | -0.13 | -0.04 | 0.000 |
| Sex |  | 0.48 | 0.22 | -0.19 | 1.16 | 0.158 |
| Affective ToM | 0.09 |  |  |  |  |  |
| Age |  | -0.05 | -0.29 | -0.07 | -0.02 | 0.000 |
| Sex |  | -0.11 | -0.10 | -0.45 | 0.23 | 0.516 |
| Education |  | 0.02 | 0.05 | -0.04 | 0.07 | 0.526 |
| Anxiety |  | -0.02 | -0.06 | -0.08 | 0.04 | 0.559 |
| Depression |  | 0.01 | 0.01 | -0.09 | 0.10 | 0.912 |
| Fluid IQ |  | 0.02 | 0.06 | -0.03 | 0.06 | 0.427 |
| Minimally adjusted model 0.08 | | | | | | |
| Age |  | -0.05 | -0.28 | -0.07 | -0.02 | 0.000 |
| Sex |  | -0.15 | -0.13 | -0.47 | 0.18 | 0.368 |
| **Emotional empathy**  Empathic concern | 0.11 |  |  |  |  |  |
| Age |  | 0.08 | 0.12 | -0.01 | 0.18 | 0.091 |
| Sex |  | 2.33 | 0.49 | 0.67 | 3.99 | 0.006 |
| Education |  | -0.08 | -0.05 | -0.32 | 0.15 | 0.488 |
| Anxiety |  | 0.25 | 0.18 | -0.00 | 0.51 | 0.051 |
| Depression |  | -0.32 | -0.18 | -0.66 | 0.01 | 0.059 |
| Fluid IQ |  | 0.19 | 0.15 | 0.02 | 0.36 | 0.031 |
| Minimally adjusted model 0.07 | | | | | | |
| Age |  | 0.06 | 0.08 | -0.04 | 0.16 | 0.273 |
| Sex |  | 2.92 | 0.61 | 1.28 | 4.56 | 0.000 |
| Personal distress | 0.16 |  |  |  |  |  |
| Age |  | 0.03 | 0.05 | -0.07 | 0.13 | 0.516 |
| Sex |  | 0.92 | 0.20 | -0.59 | 2.43 | 0.231 |
| Education |  | -0.09 | -0.06 | -0.30 | 0.12 | 0.414 |
| Anxiety |  | 0.28 | 0.21 | 0.04 | 0.53 | 0.024 |
| Depression |  | 0.31 | 0.18 | 0.02 | 0.60 | 0.036 |
| Fluid IQ |  | -0.13 | -0.11 | -0.29 | 0.02 | 0.010 |
| Minimally adjusted model 0.01 | | | | | | |
| Age |  | 0.03 | 0.04 | -0.07 | 0.13 | 0.601 |
| Sex |  | 1.11 | 0.24 | -0.42 | 2.65 | 0.154 |
| Emotional empathy total | 0.16 |  |  |  |  |  |
| Age |  | 0.12 | 0.13 | -0.01 | 0.24 | 0.067 |
| Sex |  | 3.27 | 0.52 | 1.24 | 5.30 | 0.002 |
| Education |  | -0.16 | -0.08 | -0.44 | 0.11 | 0.242 |
| Anxiety |  | 0.55 | 0.30 | 0.21 | 0.88 | 0.002 |
| Depression |  | -0.02 | -0.01 | -0.38 | 0.35 | 0.924 |
| Fluid IQ |  | 0.06 | 0.04 | -0.18 | 0.30 | 0.61 |
| Minimally adjusted model 0.07 | | | | | | |
| Age |  | 0.08 | 0.09 | -0.04 | 0.21 | 0.195 |
| Sex |  | 4.03 | 0.64 | 1.97 | 6.08 | 0.000 |

**Supplementary Table 3.**

*Spearman’s Rank Correlation Coefficients for each control variable.*

|  | M (SD) | Age | Sex | Education | Verbal IQ | Anxiety |
| --- | --- | --- | --- | --- | --- | --- |
| Age | 60.30 (6.88) |  |  |  |  |  |
| Sex |  | -0.10 |  |  |  |  |
| Education | 13.50 (2.88) | -0.07 | -0.06 |  |  |  |
| Verbal IQ | 109.44 (8.35) | **0.22** | 0.02 | **0.46** |  |  |
| Anxiety | 5.44 (3.41) | **-0.18** | 0.11 | 0.02 | -0.01 |  |
| Depression | 2.56 (2.60) | 0.09 | -0.02 | -0.10 | -0.03 | **0.47** |

*Note.* Bolded p-values are significant (*p*<0.05), sex was coded as 1 = male or 2 = female.

**Supplementary Figure 1**


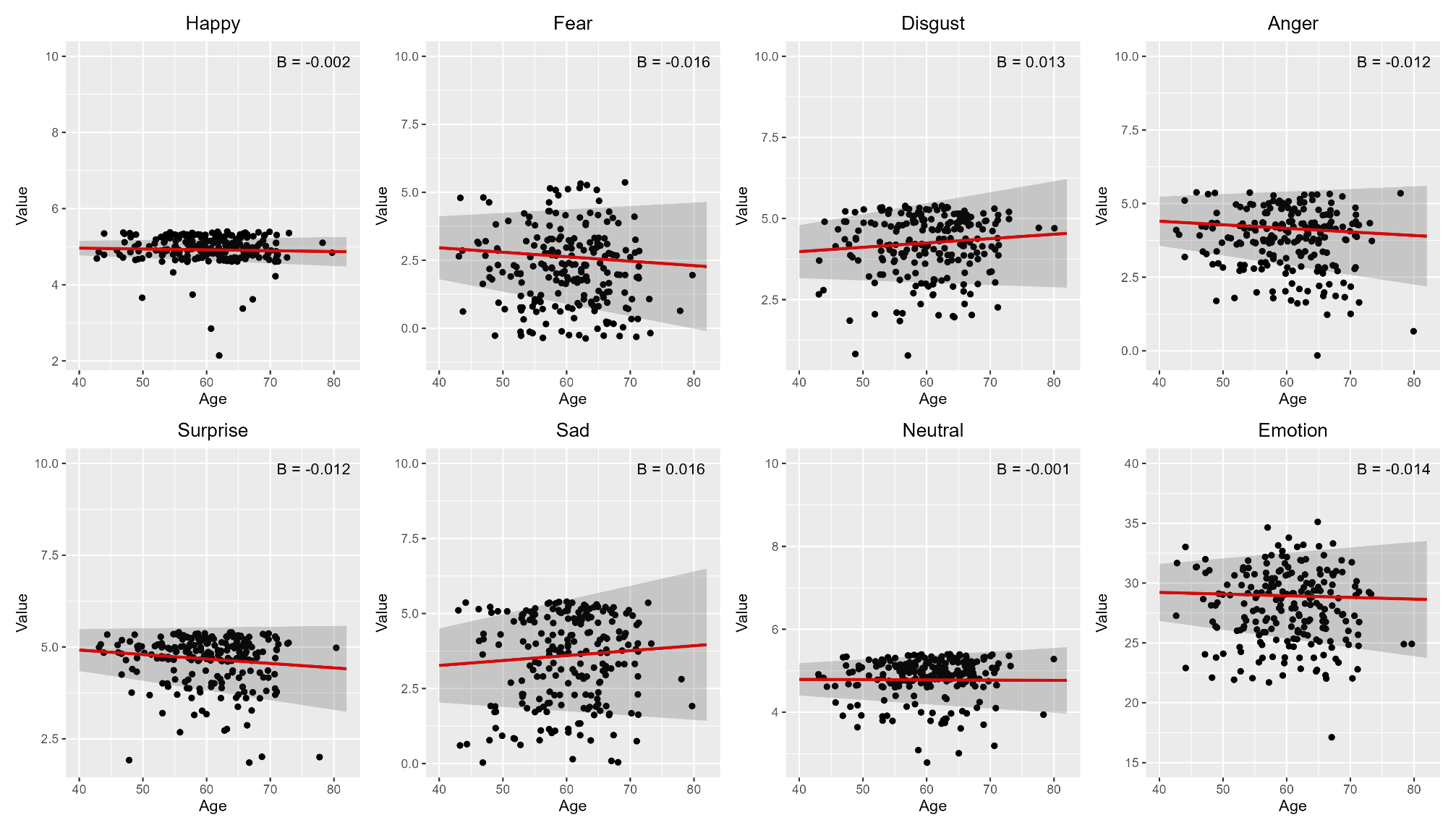
*Visual comparison of non-significant B values (p*>.017) *for age across emotion perception outcome variables. Slope of line is equal to B (the standardised coefficient). Intercept is equal to the intercept of robust multiple regression model. Shaded areas indicate 95%CI of B.*

**Supplementary Figure 2**

*Visual comparison of non-significant B values (p*>.017) *for age across emotional empathy outcome variables. Slope of line is equal to B (the standardised coefficient). Intercept is equal to the intercept of robust multiple regression model. Shaded areas indicate 95%CI of B.*

*
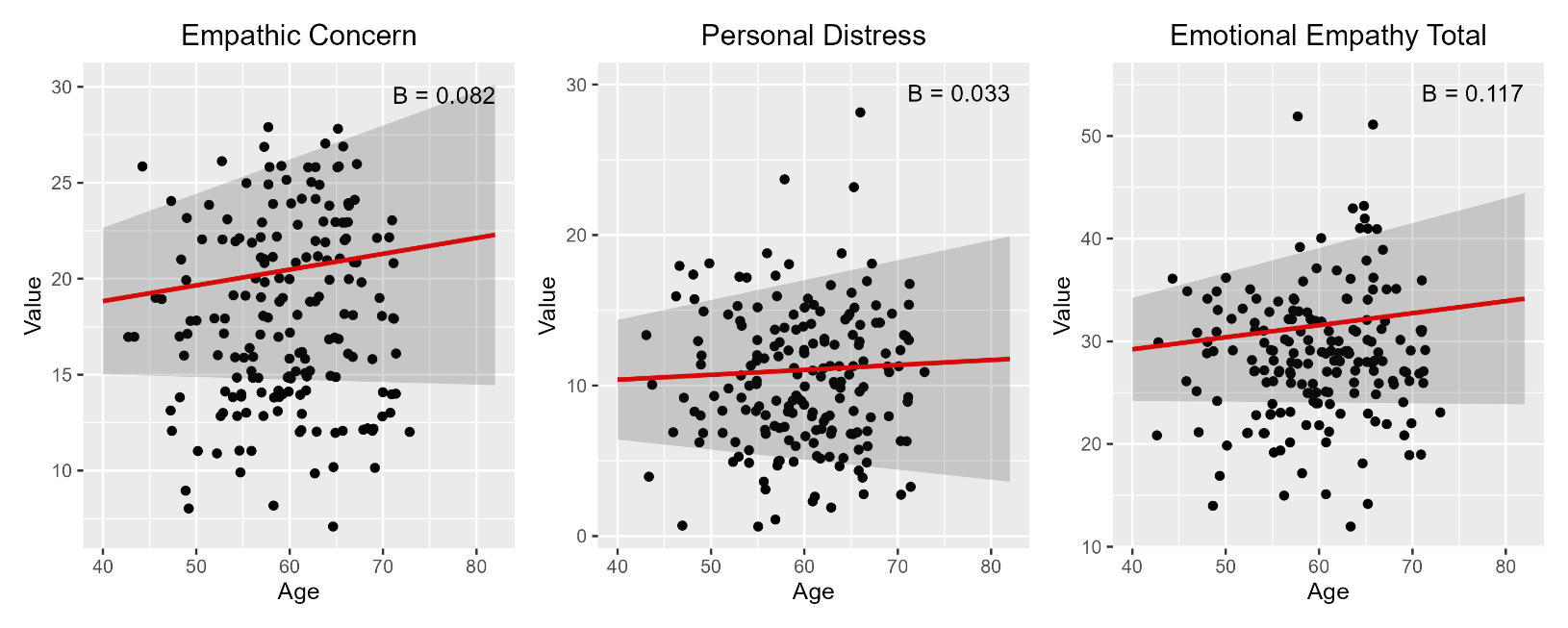
*

**Supplementary Figure 3**


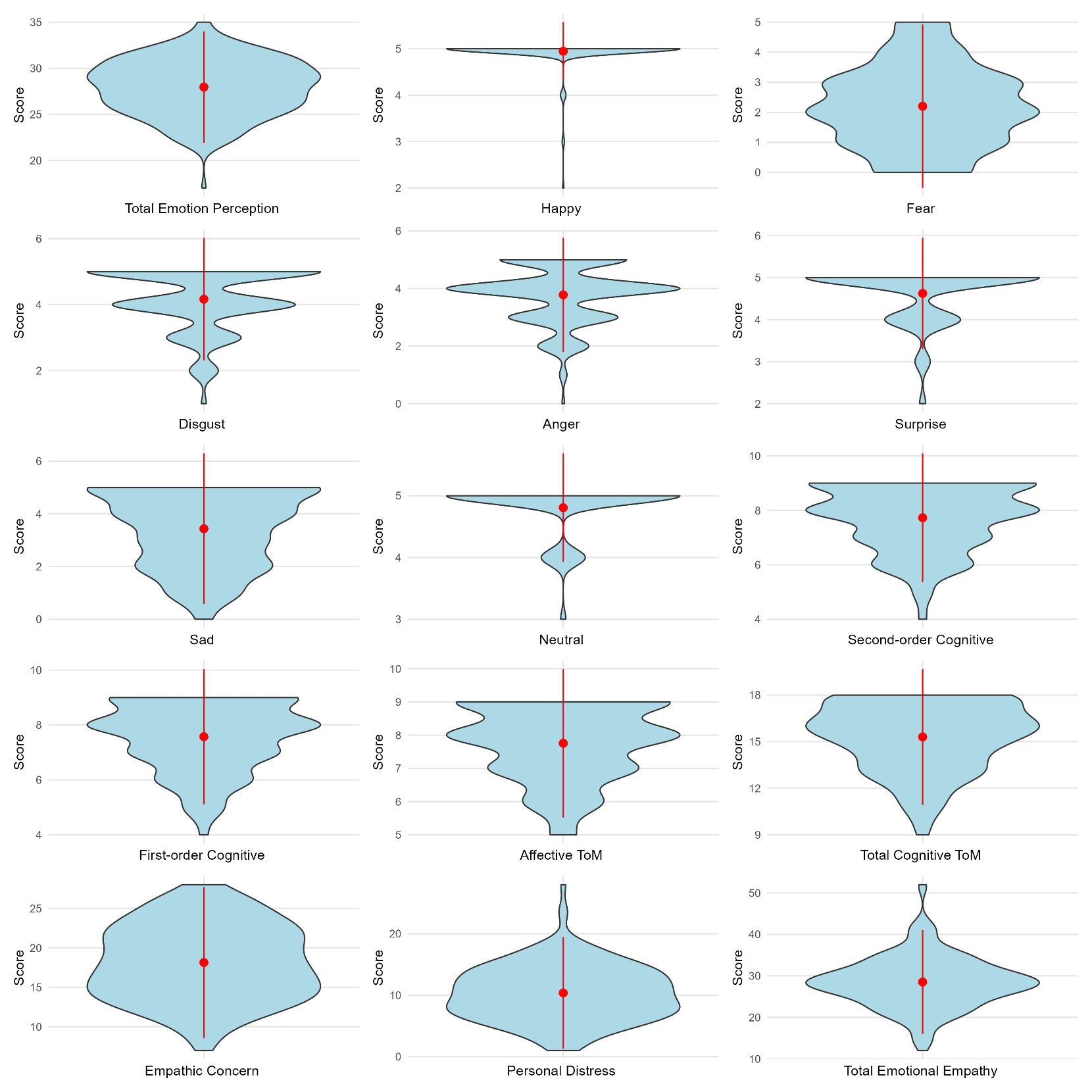
*Description*
